# Spatial complexity of character-based writing systems and arithmetic in primary school: a longitudinal study

**DOI:** 10.3389/fpsyg.2015.00333

**Published:** 2015-03-26

**Authors:** Maja Rodic, Tatiana Tikhomirova, Tatiana Kolienko, Sergey Malykh, Olga Bogdanova, Dina Y. Zueva, Elena I. Gynku, Sirui Wan, Xinlin Zhou, Yulia Kovas

**Affiliations:** ^1^InLab, Department of Psychology, Goldsmiths, University of LondonLondon, UK; ^2^Laboratory for Cognitive Investigations and Behavioral Genetics, Department of Psychology, Tomsk State UniversityTomsk, Russia; ^3^Institute of Psychology, Russian Academy of SciencesMoscow, Russia; ^4^Psychological Institute, Russian Academy of EducationMoscow, Russia; ^5^State Key Laboratory of Cognitive Neuroscience and Learning, Department of Psychology, Beijing Normal UniversityBeijing, China

**Keywords:** early arithmetic, cross-cultural, longitudinal, character-based writing system, spatial ability

## Abstract

Previous research has consistently found an association between spatial and mathematical abilities. We hypothesized that this link may partially explain the consistently observed advantage in mathematics demonstrated by East Asian children. Spatial complexity of the character-based writing systems may reflect or lead to a cognitive advantage relevant to mathematics. Seven hundered and twenty one 6–9-year old children from the UK and Russia were assessed on a battery of cognitive skills and arithmetic. The Russian children were recruited from specialist linguistic schools and divided into four different language groups, based on the second language they were learning (i.e., English, Spanish, Chinese, and Japanese). The UK children attended regular schools and were not learning any second language. The testing took place twice across the school year, once at the beginning, before the start of the second language acquisition, and once at the end of the year. The study had two aims: (1) to test whether spatial ability predicts mathematical ability in 7–9 year-old children across the samples; (2) to test whether acquisition and usage of a character-based writing system leads to an advantage in performance in arithmetic and related cognitive tasks. The longitudinal link from spatial ability to mathematics was found only in the Russian sample. The effect of second language acquisition on mathematics or other cognitive skills was negligible, although some effect of Chinese language on mathematical reasoning was suggested. Overall, the findings suggest that although spatial ability is related to mathematics at this age, one academic year of exposure to spatially complex writing systems is not enough to provide a mathematical advantage. Other educational and socio-cultural factors might play a greater role in explaining individual and cross-cultural differences in arithmetic at this age.

## Introduction

Research has shown that East Asian children on average outperform other children in mathematics (Miura, 1987; Song and Ginsburg, 1988; Stevenson and Stigler, 1992; Geary et al., 1993; Stevenson et al., 1993; Imbo and Vandierendonck, 2007; Mullis et al., 2008; OECD, 2010; Rodic et al., 2014). This advantage might partly be explained by the regular structure of the East Asian number system, as well as by the shorter pronunciation of numbers that leads to a greater digit span (Dehaene, 1997).

In our previous research we investigated whether spoken Chinese language leads to better arithmetic skills in pre-school children (Rodic et al., 2014). We assessed children from China, Russia, UK and two populations from Kyrgyzstan (Kyrgyz and Dungan), on arithmetic and other cognitive tests. As the Dungan population is ethnically similar to Chinese, speaks a form of Mandarin but uses Cyrillic (instead of character based Mandarin) as a writing system, we were able to test for the effect of the spoken language while controlling for its written aspect. Dungan children did not show any advantage in arithmetic over Kyrgyz children. This suggests that using oral Chinese, with its transparent number system and faster pronunciation of numbers, does not lead to mathematical advantage, at least for early arithmetic.

Other cognitive factors, such as spatial ability, might also play a role in the observed cross-cultural differences. For example, greater spatial complexity and increased visuo-spatial demands of Chinese reading and writing systems may lead to better mathematical performance.

Although the direction of effects and the nature of the association between spatial ability and mathematics remain unclear, they seem to be intrinsically linked. One recent genetically informative study examined the relative contribution of genetic and environmental factors to variation in spatial ability and to its relationship with different aspects of mathematics in 4174 pairs of 12-year-old twins (Tosto et al., 2014). The results suggested that, individual differences in spatial ability and different aspects of mathematics stem from both, common genetic (60%) and environmental (40%) factors. The observed correlation between spatial and mathematical ability was largely explained by overlapping genetic effects, but also overlapping environmental factors. At the level of the brain, both spatial cognition and number processing have been shown to rely on parietal lobes, especially the Intra Parietal Sulcus (Dehaene, 1997). At the behavioral level, many studies found associations between different aspects of spatial and mathematical abilities across development. For example, spatial sketchpad of working memory and mathematics performance were found to correlate (0.41) in second graders (Krajewski and Schneider, 2009). A correlation has also been observed between performance on a 3-D mental rotation task and mathematical word problem solving tasks in six graders (Van Garderen and Montague, 2003). Spatial ability has been found to correlate with mathematical ability over and above general cognitive ability in adults, both in the US (Rohde and Thompson, 2007), and China (Wei et al., 2012). Mathematically gifted adolescents perform better on spatial tasks than their non-gifted peers (Hermelin and O’Connor, 1986; Dark and Benbow, 1991).

Multiple potential mechanisms underlie the observed space-mathematics associations, from spatial representations of magnitudes on a mental number line, to spatial representations of mathematical relations, to the use of diagrams in algebraic problem solving (Geary, 1994, 1995; Hubbard et al., 2005).

It is possible that the observed advantage of representatives of East Asian cultures in mathematics can be at least partially explained by the spatial-mathematical link. Previous research indicates that spatial ability may causally contribute to mathematical learning (Rohde and Thompson, 2007; Wai et al., 2009). There is also evidence that East Asian populations show an average advantage in visuo-spatial abilities (Sakamoto and Spiers, 2014). This advantage may be related to the complexity of the character-based writing, which may either reflect or lead to superior spatial ability of some East Asian populations.

In contrast to letter-based scripts, where complexity is linear (reflected in the number of letters in a word), the complexity of Chinese characters increases with the number of elements (strokes and sub character components) packed into the same square configuration. When learning to read a Chinese character, both visual-orthographic processing and spatial analysis are essential (Tan et al., 2005). It is possible that continuous engagement in such processing leads to superior development of the relevant brain networks, which in turn leads to advantages in mathematics. In contrast, linear orthographic representations may lead to the development of the language relevant brain networks and their employment for solving mathematical problems. Support for the differential cortical number-related activity across populations was found in an fMRI study comparing native English speakers to native Chinese speakers (Tang et al., 2006). Native English speakers employed language processes for mental calculation (e.g., simple addition), while native Chinese speakers employed visuo-premotor association network for the same task.

The current study aims to investigate the spatial-mathematical link in 6–9-year-old Russian and UK children in the context of language learning over one school year. The children were assessed on a cognitive test battery measuring general skills (e.g., speed of processing), IQ, spatial ability, symbolic number understanding, non-symbolic comparison of numerosity, numerical reasoning, and arithmetic. The testing took place twice during the school year, once at the beginning and once at the end. Russian children were monolingual and began learning different second languages at the beginning of the school year. The languages included character-based systems (Chinese and Japanese) and alphabet-based systems (English and Spanish). The UK sample served as a control.

We tested the following 2 hypotheses:

(1) Spatial ability (Mental rotation) at the beginning of the academic year will predict arithmetic scores (Simple subtraction) at the end of the academic year, in both, Russian and UK 6–9 year-old children, even after controlling for IQ scores.
(2) Children who learn a second language that employs a spatially complex character-based writing system (Chinese and Japanese) will show a greater gain in mathematics and related abilities after 1 year of learning.

Although we are aware that the current design does not allow us to control for the effect of other linguistic factors, such as faster pronunciation of numbers and transparency of the number system, our previous study suggested no effect of the spoken language on arithmetic in the Dungan population (Rodic et al., 2014). In addition, the aim of our study is not to assess potential advantages of solving mathematical problems in a particular language (i.e., Russian children use Russian language for mathematical learning), but instead to test whether the process of learning and using spatially complex characters as a second language leads to some mathematically advantageous cognitive shift.

## Materials and Methods

### Participants

Seven hundred and twenty one 6–9 year-old children were recruited through primary schools in the UK and Russia. The children were tested in two waves, once at the beginning and once at the end of the 2012/2013 academic year. In the first wave of testing there were 155 UK participants from 5 schools in London (69 boys; mean age = 85 months, range 72–108); and 566 Russian participants from 15 schools across Russia (246 boys; mean age = 98.5, range = 88–104 months). In the second wave of testing, the number of participants has reduced to 145 UK participants (63 boys; mean age = 90 months, range = 80–105 months); and 438 Russian participants (185 boys; mean age = 105.8 months, range = 96–121 months). Attrition in the UK sample was mostly due to children changing schools. The substantial attrition in the Russian sample was largely due to a technical problem with the on-line test administration or access to remote samples in some regions.

The Russian participants were in the second year of their primary school education. Because Russian children start their primary education at 7 years of age (a year later than the UK children), they were inevitably older than the UK children of the same school year. In order to match the UK and Russian participants, both on their chronological age and years of education, half of the UK children were in the second year and half were in the third year of their primary education.

In the UK sample none of the children were learning a 2nd language at school before or during the year of testing. Although 30% of the sample (44 children) reported to be bi-lingual (indicated speaking languages other than English at home), none of the children used character-based writing systems.

All Russian children in the sample were monolingual and started learning the second language at school at the beginning of the school year. Out of 566 children, 379 started learning English language; 25 – Japanese; 74 – Spanish and English; and 88 – Chinese and English languages. On average, children had between 2 and 4 sessions (45 min each) of second language lessons per week. All schools were specialist language schools with enhanced language curricula. Selection into the language schools is not entirely random, although no special entry requirements are practiced and many children are enrolled on the basis of living proximity. However, parents’ willingness to enroll children into specialist language schools and belief in the children’s ability to cope with the pressures of learning extra languages can be considered as a ‘self selection’ violation to random enrolment.

The project received approval from the Ethics Committees of Goldsmiths, University of London; and Tomsk State University. Parental consent was obtained prior to data collection.

### Measures and Procedure

**FIGURE 1:**
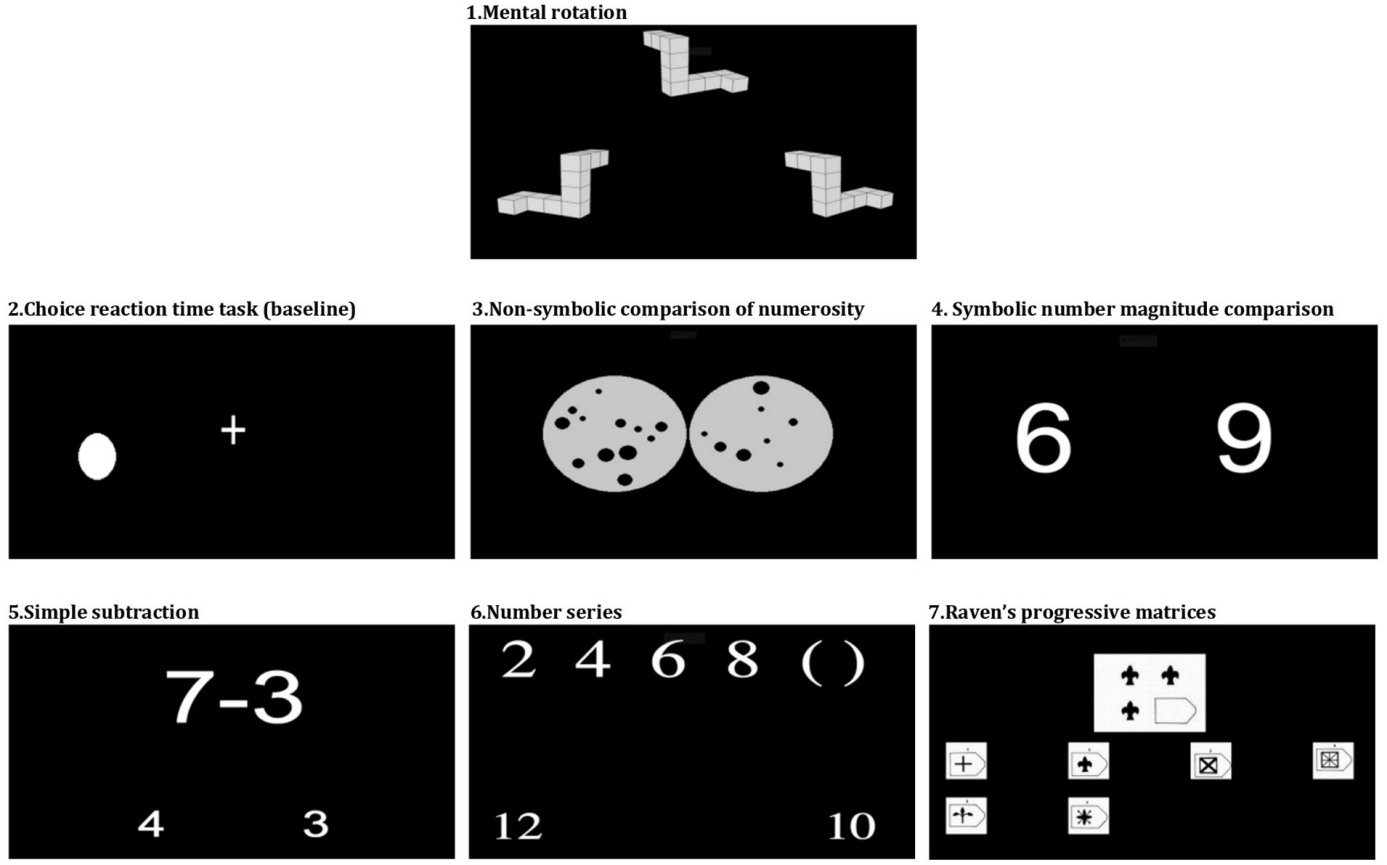
**Illustration of tasks used in the experiment, in the order of presentation**.

The battery of tests included seven on-line (www.dweipsy.com/lattice) computerized tasks (see **Figure 1**) administered in a single session at schools. The testing lasted approximately 40 min. All tests started with practice trials and were always administered in the following order: Mental rotation, Choice reaction time, Non-symbolic comparison of numerosity, Symbolic number magnitude comparison, Simple subtraction, Number series and Raven’s progressive matrices. Children indicated their responses by pressing “Q” or “P” (or corresponding Russian keys) marked with the stickers on the keyboard. For Choice reaction time, Non-symbolic comparison of numerosity and Symbolic number magnitude comparison tasks accuracy and RT (milliseconds) were recorded. For the rest of the tasks, the dependent variable was correct minus incorrect responses, correcting for guessing. The tasks are described in the following section, grouped in five categories: (1) general skills and IQ; (2) spatial ability; (3) symbolic number understanding; (4) non-symbolic number sense; (5) operating with numbers (arithmetic), and numerical reasoning. Internal validity of each measure was assessed using Cronbach’s alpha analysis. The Cronbach’s alphas, reported below separately for the two samples, are based on the first wave of data collection. The results from the second wave were highly similar.

### General Skills and IQ

*Choice reaction time* task (Butterworth, 2003) assessed accuracy and speed with which children responded to the dot appearing on the left (15 trials) or right (15 trials) side of the fixation ‘+.’ The task was time-unconstrained. The inter-stimulus interval varied randomly from 1500 to 3000 ms. Cronbach’s α = 0.65 (*N* = 154, UK sample) and Cronbach’s α = 0.87 (*N* = 555, Russia).

*Raven’s progressive matrices* (Raven et al., 1998) measured general intelligence. Participants were presented with an incomplete figure and had to identify the missing segment that would complete the figure’s intrinsically regular pattern. Children used a mouse to indicate which out of the presented six segments was the correct one. The children had 4 min to go trough as many trials as they could (80 trials in total). Cronbach’s α = 0.67 (*N* = 154, UK sample) and Cronbach’s α = 0.73 (*N* = 543, Russia).

### Spatial Ability

*Mental rotation* task (Shepard and Metzler, 1971) evaluated children’s ability to mentally rotate three dimensional images. The target image was presented on the upper part of the screen, with two possible answers presented on the left and right bottom parts of the screen. The child had to decide which of the bottom two figures was matching the figure at the top by pressing either left or right button. The matching images were rotated from 15 to 345°. Children had to select the correct answer in as many trials as they could in 3 min (180 trials in total). Cronbach’s α = 0.75 (*N* = 140, UK sample) and Cronbach’s α = 0.87 (*N* = 564, Russia).

### Symbolic Number Understanding

*Symbolic number magnitude comparison* task (Girelli et al., 2000) used a Stroop-like paradigm to assess the ability to compare numerical values of numbers. Two digits of varying sizes (1:2 size ratio) appeared simultaneously on the screen. The trials were divided into congruent, incongruent and neutral trials. In the congruent condition a numerically larger digit (e.g., 8) was also physically larger than a numerically smaller digit (e.g., 3). In the incongruent condition, three is physically larger than eight, and in the neutral condition, both digits are of the same physical size. Children had 5 s to decide which number was larger in numerical magnitude, ignoring differences in physical size. Three sessions of 28 trials each were separated by 10-s resting periods. Cronbach’s α = 0.77 (*N* = 153, UK sample) and Cronbach’s α = 0.87 (*N* = 545, Russia).

### Non-Symbolic Number Sense

*Non-symbolic comparison of numerosity* (Baroody and Ginsburg, 1990) measured non-symbolic number sense. Children had to estimate (without counting) which of the two sets of dots of varying sizes, presented simultaneously on the screen, contained more dots (36 trials, 5 s per trial). In all sets the combined area of all dots was controlled to be the same. The number of dots varied from 5 to 12; ratios were 2:3, 5:7, and 3:4. Cronbach’s α = 0.78 (*N* = 153, UK sample) and Cronbach’s α = 0.84 (*N* = 549, Russia).

### Operating with Numbers (arithmetic) and Numerical Reasoning

*Simple subtraction* task assessed early arithmetic ability. The minuends were all smaller than 18 and the differences were single-digit numbers. Two candidate answers were presented beneath the problem, one on each side of the screen. Children had to select the correct answer in as many trials as they could in 2 min (92 problems). Correct and incorrect answers were within the range of each other plus or minus 3. Cronbach’s α = 0.75 (*N* = 152, UK sample) and Cronbach’s α = 0.73 (*N* = 542, Russia).

*Number series completion* task (Smith et al., 2001) measured logical numerical reasoning. A sequence of numbers was presented on the screen (e.g., 1,3,5,7) with two additional numbers below it. The child was asked to infer the pattern of these numbers and decide which out of the two candidate answers presented below the sequence should complete the sequence (e.g., 9 or 16). The children were given 4 min to do as many sequences as they could. Cronbach’s α = 0.65 (*N* = 146, UK sample) and Cronbach’s α = 0.63 (*N* = 589, Russia).

## Results

### Growth

First, we evaluated average growth on each assessed measure over one academic year. This was done separately for the UK and Russian samples, as the two samples could not be directly compared: UK sample was heterogeneous in terms of biological age and years of schooling; the Russian children were selected from specialist language schools (see **Table 1** for mean and SDs for the raw scores for both samples on all tasks).

**Table 1:** Descriptive statistics for UK and Russian samples, for all tasks at Time 1 and Time 2.

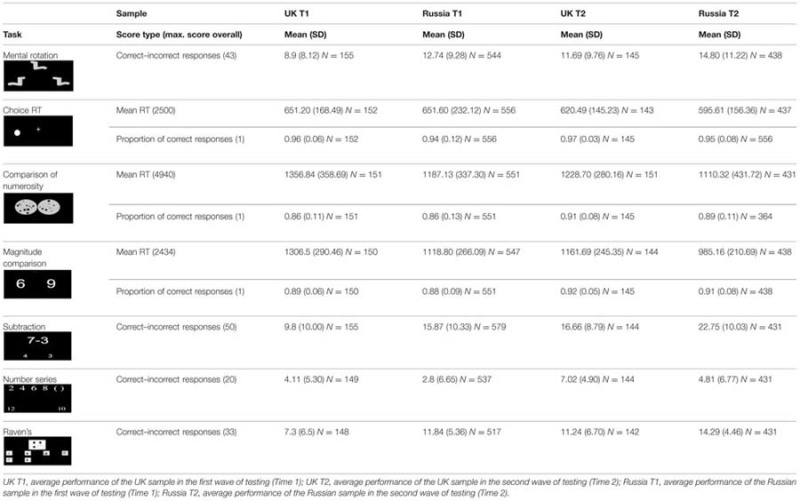

As can be seen from **Table 2**, children’s performance improved significantly for all tasks, with the exception of RT in Choice Reaction Time in the UK sample. The effect sizes of growth, obtained by means of one way repeated measures ANOVAs, ranged from 2.1% (for RT and accuracy of Choice RT task in the Russian sample) to 44% (for Simple subtraction in the UK sample).

**Table 2:** The effect sizes for growth over the school year, on all tasks for the UK and Russian samples.

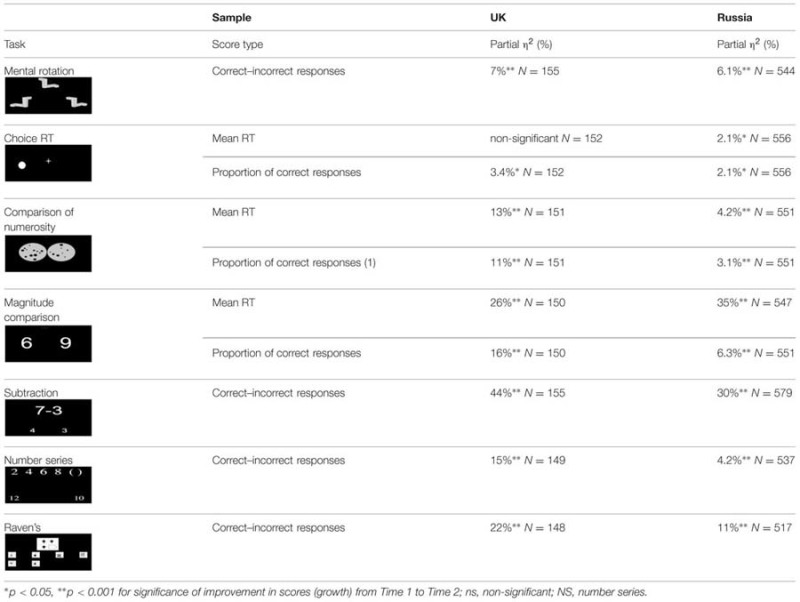

Further, we ran the between-subjects one-way ANOVAs on growth scores for each variable, calculated by subtracting the scores at time 1 from the scores at time 2, with sample as a two level factor (UK vs. Russian). The size of growth for all variables was highly similar across the Russian and the UK samples, with only one significant [but negligible, $\eta_{p}^{2}$ = 1.2%; *F*(1,525) = 6.161, *p* = 0.01] difference for the Raven’s task (see **Table 2**).

### The Relationship between Spatial Ability and Arithmetic Over Time

The cross-lag analyses, conducted on each sample separately, tested the first hypothesis regarding the longitudinal relationship between spatial ability and arithmetic, while controlling for IQ scores. This type of analysis (described below) evaluates associations between the two variables over time, while controlling for stability of each measure over time and for associations between the two measures at the same time.

#### Russian Sample

Before conducting the cross-lagged analyses, a correlation matrix was obtained and inspected to check for longitudinal associations, as well as associations between Mental rotation and Subtraction. Correlations between time 1 and time 2 assessments were moderate, both for Mental rotation (*r* = 0.507) and Subtraction (*r* = 0.498), indicating relative stability of measures over time. A modest relationship between the Mental rotation and Subtraction was found at both assessments waves (*r* = 0.221 at time 1 and *r* = 0.275 at time 2). Correlation between Mental rotation at time1 and Subtraction at time 2 was slightly higher (*r* = 0.277) than that of Subtraction at time 1 and Mental rotation at time 2 (*r* = 0.137).

Next, the cross-lag structural equation modeling (Campbell, 1963), was utilized to investigate the longitudinal relationship between spatial ability (Mental rotation) and early arithmetic (Subtraction). This type of analysis can investigate causal ordering of variables by estimating three types of relationships: (1) autoregressive paths which assess within-construct stability by estimating the correlation between two assessments of the same variable (e.g., Mental rotation at time 1 and time 2); (2) contemporaneous relationship between the two measures at the same assessment wave (e.g., Mental rotation at time 1 and Subtraction at time 1); and (3) cross-lagged relationship which estimates the extent to which scores for one variable at time 1 predict unique variance in the other variable at a later time (e.g., Mental rotation at time 1 and Subtraction at time 2), while controlling for autoregressive and contemporaneous associations. Further, we included the Raven’s scores at time 1 as a covariate in order to control for IQ on both measures at both times.

**FIGURE 2:**
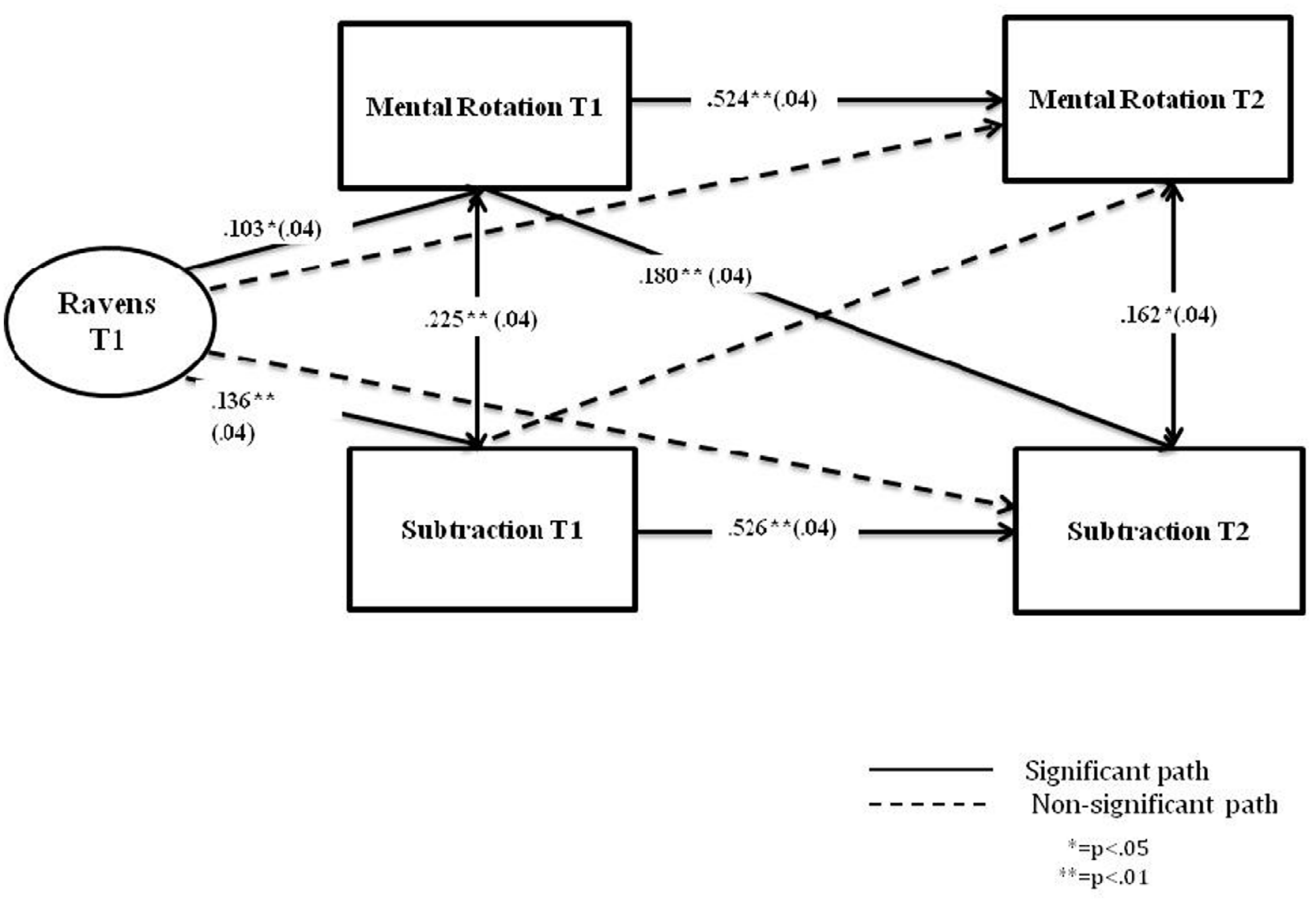
**Cross-lagged analysis of mental rotation and subtraction over one academic year, accounting for IQ (Russian sample)**.

**Figure 2** shows standardized path coefficients for the longitudinal relationship between spatial ability and arithmetic. The full model, which included the cross-lagged associations, was found to fit the data better (AIC = 4825.61), than the model excluding those associations (AIC = 4837.52). The non-significant paths were then dropped from the cross-lagged model until the best fitting model was achieved: χ^2^ (3) = 6.35, *p* = 0.098, RMSEA = 0.046, CFI = 0.990, TLI = 0.966, SRMR = 0.024 (*N* = 527). The best fitting model suggests the direction of the relationship from spatial ability to later arithmetic and not vice versa. The standardized paths are shown in **Figure 2**. Significant paths were: the cross-lagged path from Mental rotation at time 1 to Subtraction at time 2 (β = 0.180, SE = 0.04, *p* < 0.001); the contemporaneous paths between Mental rotation and Subtraction at both, time 1 (β = 0.225, SE = 0.04, *p* < 0.001) and time 2 (β = 0.162, SE = 0.04, *p* = 0.002); and the autoregressive paths for both, Mental rotation (β = 0.524, SE = 0.04, *p* < 0.001) and arithmetic (β = 0.526, SE = 0.04, *p* < 0.001). The paths from the covariate (Raven’s) were significant for Mental rotation at time 1 (β = 0.103, SE = 0.04, *p* = 0.018); and Subtraction at time 1 (β = 0.136, SE = 0.04, *p* = 0.002).

#### The UK Sample

Correlations between time 1 and time 2 assessments were moderate, both for Mental rotation (*r* = 0.434) and Subtraction (*r* = 0.575), indicating relative stability of measures over time. A modest relationship between the Mental rotation and Subtraction was found at both assessments waves (*r* = 0.185 at time 1 and *r* = 0.195 at time 2). Correlation between Mental rotation at time 1 and Subtraction at time 2 was not significant, while Subtraction at time 1 and Mental rotation at time 2 were modestly correlated (*r* = 0.209).

Next, cross-lag analysis was conducted in order to investigate the relationship between the spatial ability (Mental rotation) and early arithmetic (Subtraction) while accounting for the IQ scores.

**FIGURE 3:**
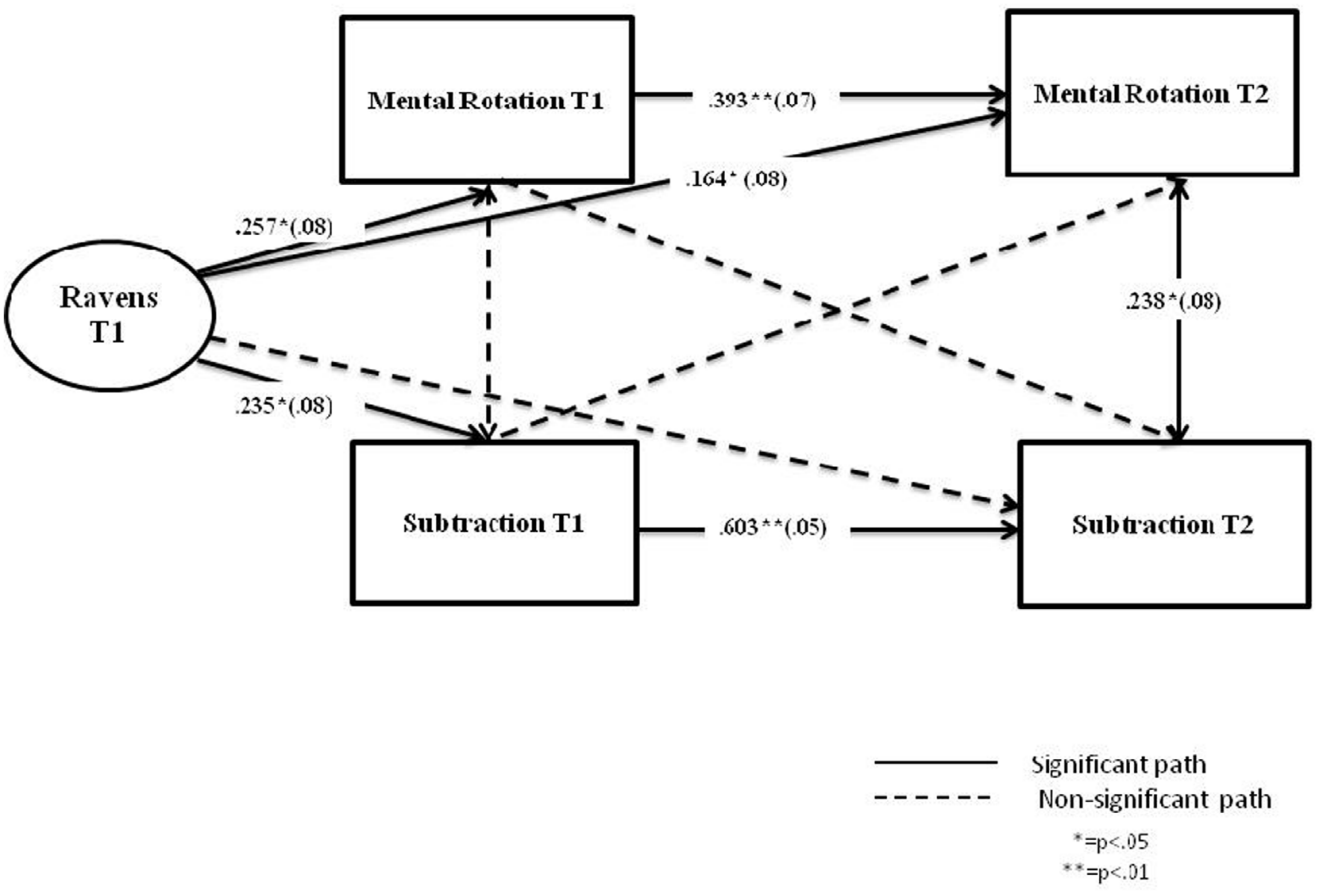
**Cross-lagged analysis of mental rotation and subtraction over one academic year, accounting for IQ (UK sample)**.

**Figure 3** shows standardized path coefficients for the longitudinal relationship between spatial ability and arithmetic in the UK sample. The model excluding the cross-lag associations was found to fit the data better (AIC = 1428.342) than the full model which included those associations (AIC = 1425.781). The non-significant paths were then dropped from the model. The model in **Figure 3** fitted the data very well: χ^2^ (4) = 2.618, *p* = 0.062; RMSEA <0.001; CFI = 1.00; TLI = 1.031; SRMR = 0.036 (*N* = 144). In the UK sample, the significant paths included: the contemporaneous path between Mental rotation and Subtraction at time 2 (β = 0.238, SE = 0.08, *p* = 0.004); and the autoregressive paths for both Mental rotation (β = 0.393, SE = 0.07, *p* < 0.001) and Subtraction (β = 0.603, SE = 0.05, *p* < 0.001).

The paths from the covariate (Raven’s) were significant for Mental rotation at time 1 (β = 0.257, SE = 0.08, *p* = 0.001); Mental rotation at time 2 (β = 0.164, SE = 0.08, *p* = 0.035); and Subtraction at time 1 (β = 0.235, SE = 0.08, *p* = 0.003).

### Second Language Acquisition Effects on Cognitive Skills and Arithmetic

The second hypothesis, addressing the effects of second language learning on arithmetic and related skills, was investigated in the Russian sample. The sample was split into four groups based on the different languages that children learn at school (i.e., English, Japanese, Spanish and English and Chinese and English). **Table 3** shows the descriptive statistics for the four language groups at both times.

**Table 3:** Descriptive statistics for the Russian sample language groups, for all tasks at both times.

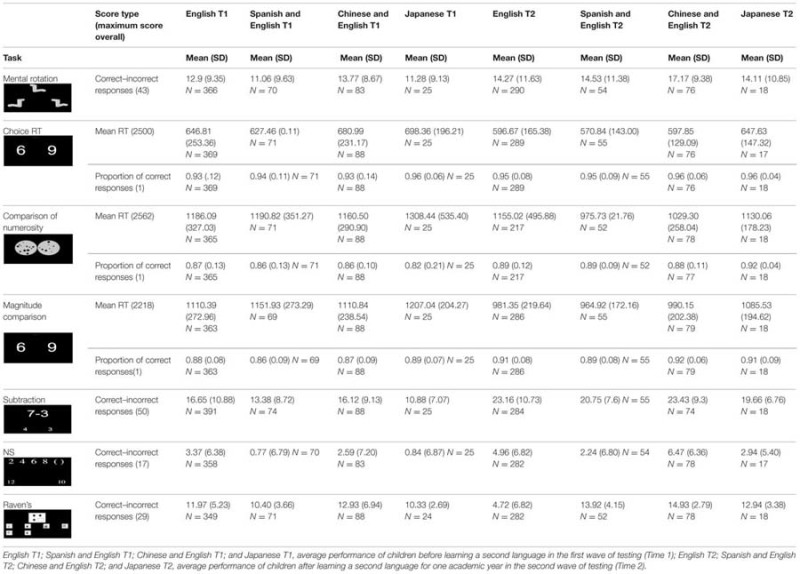

One-way ANOVAs were employed to test for differences on all tasks between the four groups at the beginning of the year (time 1). Despite the differences in sample sizes (379 for English; 25 for Japanese; 74 for Spanish; 88 for Chinese), performance at baseline was overall similar across the four groups. No significant differences between the groups were found for RT and accuracy of Choice reaction time; RT and accuracy of Symbolic number magnitude comparison; RT and accuracy of Non-symbolic comparison of numerosity; and Mental rotation (correct minus incorrect responses score). For the remaining three tasks significant (*p* < 0.05), but very small ($\eta_{p}^{2}$ = 2.1–3.2%, *p* < 0.05) differences were found (details available from the authors). Only one violation to equal variance was found (for the Raven’s task), but the differences in variance were negligible (as suggested in Field, 2009).

Next, to test for the effect of the language learnt at school on task performance at the end of the year, we conducted ANCOVA, including the performance on the task at the baseline time 1 as a covariate. The only significant effect of language was on the Number series completion task [*F*(3,399) = 4.063, *p* = 0.007], with Chinese/English learning group slightly ($\eta_{p}^{2}$ = 3%), outperforming Japanese (*p* = 0.021) and Spanish/English (*p* = 0.001) learning groups.

## Discussion

The study set out to investigate the relationship between spatial ability and mathematical performance. First, we assessed the cross-lag relationship between spatial ability (Mental rotation) and arithmetic (Simple subtraction). The significant positive link from spatial ability to later arithmetic was found only in the Russian sample. This finding is similar to what was previously found with 18-year-old students from the US whose spatial ability predicted mathematical portion of Scholastic Aptitude Tests (SATs) even after controlling for IQ (Rohde and Thompson, 2007). The smaller number of participants and the larger standard errors in the UK sample indicated possibility of insufficient power to detect any effects that might have existed in this sample. Further, as spatial ability is a complex multifactorial domain, our findings may not extend beyond the relationship between 3-D mental rotation ability and arithmetic. Future studies with tasks measuring different aspects of spatial ability (e.g., spatial memory or navigation) are needed to assess whether different aspects of spatial ability have different relation to arithmetic.

Differences between the two samples may also indicate that the relationship between spatial ability and mathematical ability may develop differently in different cultures. However, as the UK children were between 12 and 15 months younger than the Russian children at both waves of testing, the differences could also reflect developmental processes. Future research is needed to confirm the generalizability of our finding to different populations and at different ages.

Second, owing to the access of special sampling of the Russian sample we were able to investigate whether acquisition and usage of the character-based writing system, within 1 year, could lead to a better performance in arithmetic and other cognitive skills in 6–9-year children. There were no noticeable differences between the language groups on any of the tasks at the baseline time 1. At time 2 no significant differences in performance emerged across the language groups for most tasks. The only task that showed significant, although small effect (3% of the variance) was the Number series completion task, even after controlling for the performance at the baseline. The children who learnt Chinese/English showed a small advantage over those who learnt Japanese and Spanish/English. Because these children showed the biggest improvement in this mathematically related task over 1 year, there is some indication that learning the Chinese language may positively influence mathematical reasoning. As the children were learning mathematics in Russian and were not tested in Chinese, oral advantage of Chinese language is unlikely to explain the observed advantage. It is possible that the usage of the spatially complex character-based writing system indeed plays a role in the observed advantage in mathematical ability, as suggested by our hypothesis. The lack of advantage in children who learnt Japanese, which also required learning the character-based writing system, could be due to the very small sample size of this sample (*N* = 25), but further research is needed in order to test this.

Overall, there was a significant improvement over one academic year, in both samples on all tasks. The biggest improvement in both samples (Russian = 30% and the UK = 44%), was seen for the Simple subtraction task. This is not surprising as this ability was explicitly taught to the pupils throughout the year. Furthermore, although the UK sample demonstrated bigger growth on most tasks overall, the only significant difference between samples was found on Raven’s task, with UK children showing bigger growth. This finding suggests that the developmental trajectory of mathematically relevant skills is similar for both samples.

The writing system is likely to be only one of many factors contributing to the advantage of East Asian children in mathematics. As discussed earlier, cultural ethos, parental support, frequent practice and the Confucian values that place high value on effort and academic success (Leung, 2001) – may all contribute independently.

Another possible explanation for the lack of the effect of learning a spatially complex character-based writing system is that our sample was too young. Previous studies suggested that mathematical advantage in children with better spatial skills was due to them employing spatial representations to solve mathematical problems (Geary, 1994, 1995). This skill comes with more experience and might not be used by the children in early primary school. In order for children to employ such strategies, more mathematical experience and explicit teaching of these strategies may be needed. Investigations with older children are required to explore these possibilities. Additionally, the usage of spatial strategies might not be useful for simple arithmetic. For the advantage in arithmetic in this early stage other factors might play a bigger role, such as the fast pronunciation of numbers (Geary et al., 1993) and regularity of number systems. Using spatial strategies as suggested by Geary (1995) might begin to play a role with more advanced mathematical problem solving and geometry, which is taught later in formal education.

The study had several limitations. Several tasks lacked sensitivity as they turned out to be too easy (e.g., Non-symbolic comparison of numerosity) or too difficult (e.g., Number series completion). It is possible that more sensitive tasks would yield some significant differences between the language groups. Further research with more sensitive tasks is needed to better address these issues.

Another limitation, is that the length of the period in which children were exposed to learning the character-based writing system might not have been sufficient enough for the development of greater spatial and, consequently, greater mathematical skills. It is likely that a longer and more intensive exposure (more than 3 h a week) is needed for an effect to emerge. In addition, also it is of course possible that the mathematical advantage of Asian populations is not influenced by the usage of character-based writing system, but reflects a particular distinctive cognitive feature that has led to the invention of this complex system in the first place.

Finally, the sample sizes of our language groups were very different, ranging from 25 participants for the Japanese language group to 391 participants for the English language group. The fact that Japanese language group consisted of only 25 participants at time 1 and 18 at time 2 could have significantly decreased a chance of detecting any true effects of learning that language.

## Conclusion

Despite curricular and other sample differences, the rate of learning on all tasks over one academic year was very similar for the UK and Russian children. In line with previous research, spatial ability predicted arithmetic in the Russian sample longitudinally and beyond intelligence scores. We extended previous literature by testing whether the acquisition of spatially complex character-based writing system could lead to better performance in maths, due to the established relationship between the spatial ability and mathematics. Only a small effect (3%) of learning Chinese as a second language was found on mathematical reasoning. Our findings suggest that despite the importance of spatial ability for mathematics, one academic year of increased spatial processing through exposure to spatially complex writing systems might not be enough to provide a mathematical advantage. Longer periods of exposure might be needed for it to have a positive effect on mathematics. Further cross-cultural longitudinal research is needed to identify specific cognitive, cultural, educational, linguistic and genetic influences on mathematical learning.

## Conflict of Interest Statement

The authors declare that the research was conducted in the absence of any commercial or financial relationships that could be construed as a potential conflict of interest.
